# My Encounters and Progress with Minimally Invasive Surgery

**DOI:** 10.14789/ejmj.JMJ25-0034-P

**Published:** 2025-12-05

**Authors:** KAZUHIRO SAKAMOTO

**Affiliations:** 1 Department of Coloproctological Surgery, Juntendo University Faculty of Medicine, Tokyo, Japan; 1 Department of Coloproctological Surgery, Juntendo University Faculty of Medicine, Tokyo, Japan; 2 Department of Coloproctological Surgery, Juntendo University Graduate School of Medicine, Tokyo, Japan; 2 Department of Coloproctological Surgery, Juntendo University Graduate School of Medicine, Tokyo, Japan

**Keywords:** colorectal cancer, laparoscopic surgery, robotic surgery, minimally invasive surgery

## Abstract

After graduating from Juntendo University in 1984, I joined the First Department of Surgery at Juntendo University in 1986 after two years of surgical training. When I started my career as a gastrointestinal surgeon, the only surgical treatment approach was the “open abdominal method”, which involves making a large incision in the abdomen. At that time, I did not anticipate the development of new techniques. In the late 1980s, “laparoscopic surgery” using a laparoscope and endoscopic forceps with a small wound through a port was introduced. At our hospital, laparoscopic surgery for cholelithiasis and colorectal cancer was introduced in 1991 and 1993, respectively. Laparoscopic cholecystectomy has rapidly become popular worldwide and has become the gold standard surgical procedure in Japan. With improvements in laparoscopic surgical techniques, laparoscopic surgery for colorectal cancer has evolved into a less invasive technique (reduced port surgery) and more complicated procedure.

While one would think there would be no further procedural changes in surgical treatment, another wave emerged, that of robotic surgery. A surgically assisted robotic system was approved by the Food and Drug Administration (FDA) in 2001. In Japan, the use of this system was covered by government insurance in 2012 for prostatectomy. Robotic surgery for prostatectomies was initiated at our hospital in 2013. The use of robotic surgery for colorectal cancer surgery was initiated as a clinical trial in 2015. It was covered by government insurance in 2018, and by Mar. 2025, we had performed approximately 270 robotic surgeries. It has been a great asset and very valuable experience for me to be involved in the launch of two minimally invasive surgical techniques, “laparoscopic surgery” and “robotic surgery,” during my 41 years of practice as a surgeon in Juntendo, from the time when only “open surgery” was available.

## Introduction

I graduated from the Juntendo University Faculty of Medicine in 1984; it has been almost 41 years since then. Afterward, I began my career as a gastrointestinal surgeon at Juntendo University Hospital. Here, I will reflect on my experiences with minimally invasive surgery, including laparoscopic and robotic procedures, and my professional development from when I joined the First Department of Surgery, until I retired in Mar. 2025 as a professor of the Department of Coloproctological Surgery.

## Encounter with laparoscopic surgery

**Figure 1:**
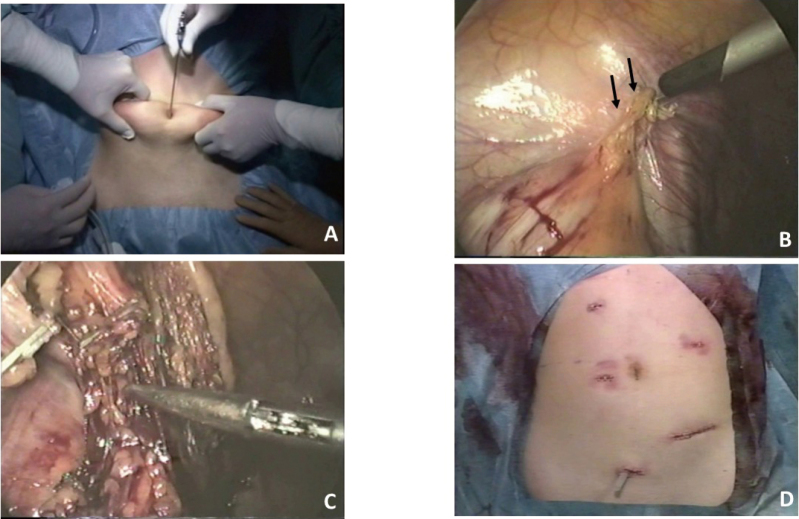
Surgical findings of the first case with an early rectosigmoid colon cancer A: Pneumoperitoneum using a pneumoperitoneum needle (the Veress needle) B: Lateral peritoneal attachments (arrows) are mobilized using a medial-to-lateral approach. C: The sigmoid mesocolon is circumferentially mobilized. D: Postoperative abdominal findings

The first laparoscopic cholecystectomy (LC) was performed by Mouret et al. in France in 1987^1)^. Subsequently, LC has rapidly become popular worldwide. In Japan, Yamakawa et al. first performed the procedure in 1990^2)^, and it was performed at Juntendo University Hospital in 1991. On the other hand, laparoscopic colorectal resection for colorectal cancer (CRC) was first reported by Jacobs et al. in 1992^3)^. In Japan, this procedure was performed by Watanabe et al. in 1992^4)^ and Kobayashi et al. at Juntendo University Hospital in 1993^5)^. At that time, the laparoscopic image on the monitor was dark and the target organ could only be recognized when closely approached. Furthermore, there were no atraumatic forceps that could grasp the intestinal tissues during laparoscopic colorectal resection. Forceps used in laparoscopic cholecystectomy were also used in the procedure (Figure 1).

**Figure 2:**
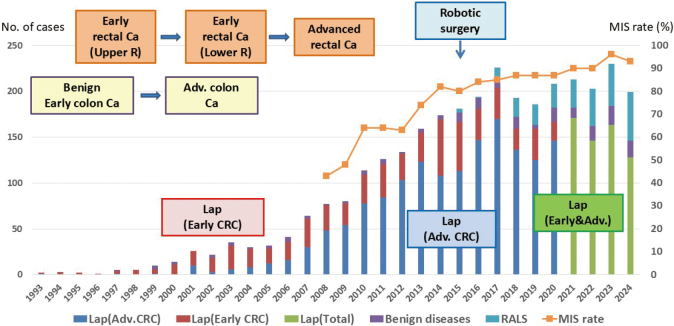
Change in laparoscopic and robotic surgery for colorectal diseases in our department

**Figure 3:**
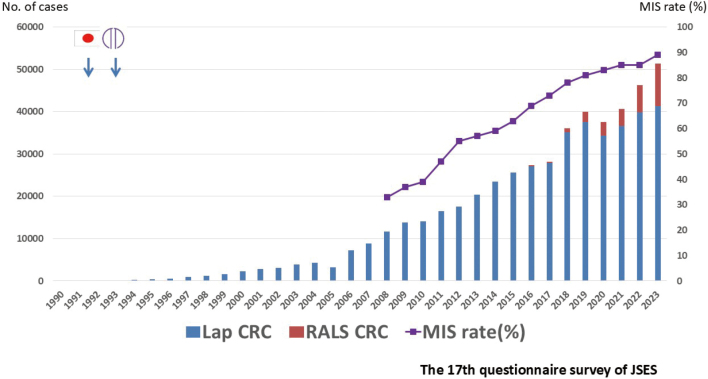
Annual number of minimally invasive surgeries for colorectal cancer in Japan

Subsequently, surgical instruments such as atraumatic forceps and endoscopic staplers have gradually improved. Initially, the number of laparoscopic surgeries performed in patients with colorectal disease in our department was low, because laparoscopic surgery was performed primarily for early stage colon cancer and benign colon diseases (Figure 2). According to the 17th Nationwide Survey of Endoscopic Surgery conducted by the Japanese Society for Endoscopic Surgery (JSES)^6)^, the number of laparoscopic surgeries performed in Japan was small in the 1990s (Figure 3).

**Figure 4:**
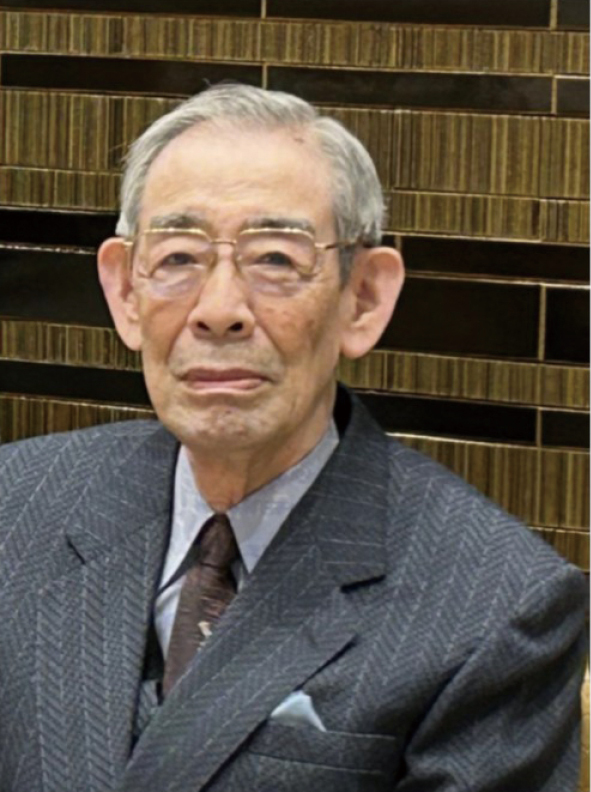
Professor Toshiki Kamano

**Figure 5:**
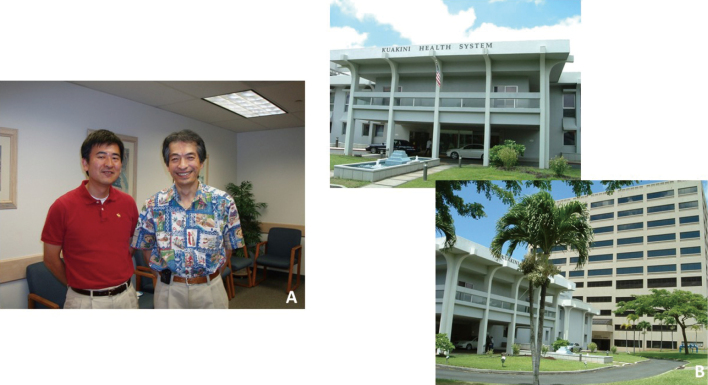
Professor Junji Machi at University of Hawaii (A) and Kuakini Hospital in Hawaii (B)

**Figure 6:**
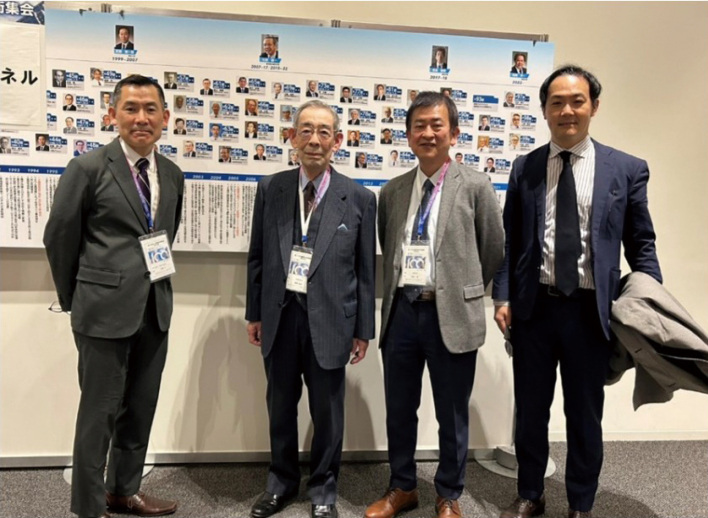
The memorial photo with Professor Toshiki Kamano in front of the memorial board at the 100th meeting of the Japanese Society for Cancer of the Colon and Rectum (JSCCR) on Jan 26, 2024, in Japan

In 2003, the Department of Surgery was reorganized. The First and Second department surgery were divided into upper gastrointestinal (GI), lower GI, hepatobiliary, pancreatic, and breast-endocrine surgery. Dr. Toshiki Kamano was appointed the first professor of the Department of Coloproctological Surgery (Figure 4), and I became a lecturer focusing on the surgical treatment of colorectal diseases. During this time, I had the opportunity to study at the Kuakini Hospital in Hawaii for a short period under the care of Professor Junji Machi of the University of Hawaii (Figure 5). I researched and reported on the clinicopathological differences between Japanese Americans in Hawaii and native Japanese individuals in Japan with CRC^7, 8)^. After completing six months studying abroad, I returned to my department in 2005. In the year I returned to Japan, I assisted with the 63rd meeting of the Japanese Society for Cancer of the Colon and Rectum (JSCCR), organized by Professor Kamano. I had the opportunity to interact with leading Japanese colorectal surgeons involved in the "Colorectal Cancer Classification Protocol." The photo was taken in front of the memorial board at the 100th JSCCR meeting (Figure 6).

Laparoscopic colorectal resection has been performed in Japan for approximately 34 years. According to the 17^th^ Nationwide Survey of Endoscopic Surgery by the JSES^6)^, the percentage of laparoscopic colorectal surgeries gradually increased, surpassing 50% in 2012. Additionally, robotic surgery was added to the tally in 2018, and the minimally invasive surgery (MIS) ratio, which included both laparoscopic and robotic surgeries, increased to 89% (Figure 3). Since the introduction of laparoscopic colorectal surgery in our department, we have gradually expanded the indications for surgery and increased the number of MIS procedures performed (Figure 2). Based on my experience, I will describe these two major waves of "laparoscopic surgery" and "robotic surgery."

## My progress with laparoscopic surgery

When laparoscopic surgery was first introduced, basic and clinical studies were conducted in a surgical environment. This environment was not previously available in open laparotomy. The studies examined patient changes associated with carbon dioxide insufflation, its effects on metastasis, safe surgical position, and port insertion^9, 10)^.

We conducted a clinical study to compare the effects of laparoscopic and open surgery on free cancer cells and perioperative patient stress. We examined carcinoembryonic antigen (CEA) mRNA positivity in the peripheral blood during the perioperative period. There was an increase in CEA mRNA positivity immediately after surgery in both groups; however, the difference was not significant. Laparoscopic surgery has been suggested as equivalent to open surgery with regard to free cancer cells^11)^. Regarding perioperative patient stress, salivary chromogranin A (CgA), serum interleukin-6 (IL-6), and C-reactive protein (CRP) levels were significantly lower in the laparoscopic surgery group than in the open surgery group. However, there were no differences in the derivatives of the Reactive Oxygen Metabolite Test (d-ROMs Test) findings between the two groups. Therefore, the blood data could not demonstrate the minimally invasive nature of laparoscopic surgery^12)^.

### Changes in upper limb fixation devices

**Figure 7:**
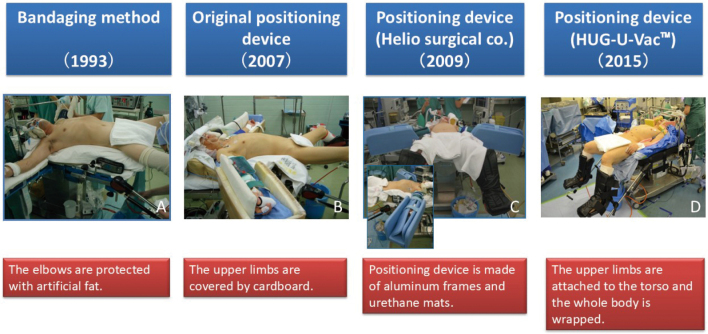
Transition of upper limb positioning devices A: The elbows are protected with artificial fat to prevent nerve compression. B: The upper limbs are covered by cardboard to prevent the upper limbs from falling. C: Positioning devices are made of aluminum frames and urethane mats. D: The device (HUG-U-Vac™) attaches the upper limbs to the torso and encases the entire body.

At the beginning of the laparoscopic procedure, the patient's upper limbs were fixed to arm boards with arm straps. After the drapes were removed at the end of the operation, the patient's forearms fell off the arm board, resulting in postoperative upper extremity neuropathy. Subsequently, nurses in the operating room made upper limb fixation devices from cardboard and polyurethane^13)^. In 2009, we commissioned a company to manufacture a sturdy upper limb fixation device using aluminum frames and urethane mats. However, it was too large and got in the way of the surgeon and the assistant. It was not until 2015 that we were able to immobilize both the upper limbs on the torso (Figure 7).

### Advances in laparoscopic surgery

Since 2003, the indications for laparoscopic surgery for CRC have gradually expanded to include advanced colon cancer and early stage lower rectal cancer (Figure 2). After Professor Kamano retired in 2006, the safety of laparoscopic surgery was verified^14)^. I also provided technical guidance for endoscopic surgery in our department and worked to increase the number of technically certified surgeons in the JSES Endoscopic Surgical Skill Qualification System.

**Figure 8:**
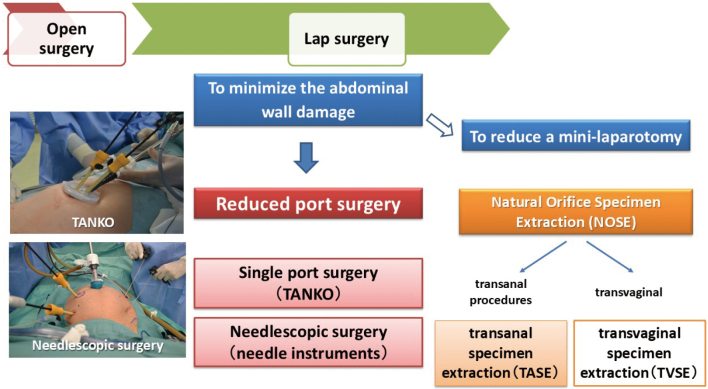
The evolution of laparoscopic surgery with a focus on minimally invasive procedures

I became a professor in 2009, and since then, the evolution of laparoscopic surgery has continued in two directions. The first is the concept of " reduced port surgery (RPS) ", which reduces the invasiveness of the abdominal wall through the ports (Figure 8). RPS includes single-port surgery, which reduces the number of ports to one, and needlescopic surgery, which uses fine forceps to make the port holes smaller^15-17)^.

### The evolution of laparoscopic surgery with a focus on minimally invasive procedures

**Figure 9:**
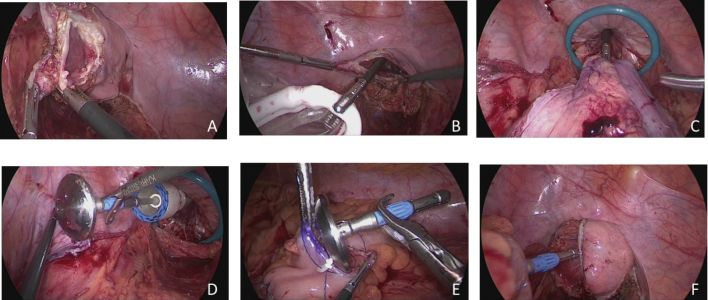
Totally laparoscopic sigmoidectomy with TASE A: After the sigmoid colon is transected on both sides, the transected rectal stump is opened. B: The wound retractor is pulled out of the anus and placed in the rectal lumen. C: The resected specimen was transanally extracted from the abdominal cavity. D: The anvil was intracorporeally attached to the stump of the proximal sigmoid colon. E: The anvil was inserted into the abdominal cavity via the anal extraction route. F: After the rectal stump is closed using an endoscopic linear stapler, colorectal anastomosis is performed using a double-stapling technique.

Natural Orifice Specimen Extraction (NOSE) involves removing a surgical specimen through a natural orifice instead of creating a small incisional abdominal wound. The technique was first reported in 1993^18)^. NOSE can be performed through the anus or the vagina. Transanal specimen extraction (TASE), in which resected bowel specimens are removed through the anus, is a reasonable extraction method for CRC. We have performed TASE for additional bowel resection after endoscopic resection in cases of early stage left-sided CRC^19, 20)^ (Figure 9).

### The evolution of laparoscopic surgery with a focus on complicated procedures

**Figure 10:**
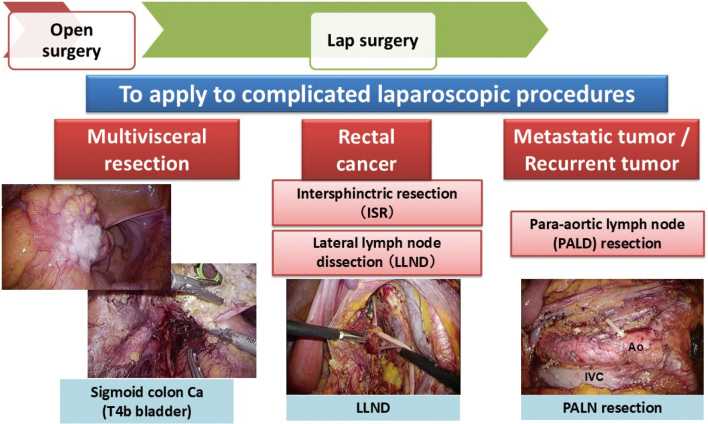
The evolution of laparoscopic surgery with a focus on complicated procedures

The other direction is the evolution toward more complex and challenging laparoscopic surgeries (Figure 10). One of these complicated procedures is the multivisceral resection of adjacent organs for locally advanced colon cancer. In a review of 65 cases of cT4 colon cancer treated with laparoscopic surgery in our department between 2010 and 2022, the abdominal wall and retroperitoneum were the organs most frequently resected (33 cases), followed by the bladder (12 cases) and the small and large intestines (11 cases). The open conversion rate was 18.5%, and 26 patients (40%) had pathological pT4b. The median postoperative hospital stay was 11 days, and operations were performed safely without any reoperation^21)^. Complicated procedures for rectal cancer (RC) include intersphincteric resection (ISR), also called ultimate sphincter-preserving surgery, and lateral lymph node dissection (LLND) for locally advanced RC. We conducted a multicenter study to verify the safety of ISR in stage I rectal cancer^22)^. We also participated in a multicenter study on anal function in laparoscopic surgery, including ISR, for lower RCs located within 5 cm of the anal verge^23, 24)^. Postoperative anal function temporarily worsened 3 months after surgery but gradually recovered and spontaneously returned to an acceptable level within 3 years. The anal function was significantly better after LAR than after ISR^25)^.

The third complicated procedure is laparoscopic surgery for recurrent cancer or distant metastasis. We have encountered cases of dissection of metachronous para-aortic lymph node metastases after primary tumor resection^26)^ as well as simultaneous operation.

Thus, we expanded the indications for laparoscopic surgery to include a variety of CRCs and accumulated a significant number of cases. Initially, I thought there would be no further changes in surgical treatment; however, another wave emerged. This was robotic surgery.

## Encountering robotic surgery

The surgically assisted robotic system was approved by the U.S. Food and Drug Administration (FDA) in 2001. In Japan, government insurance began covering prostatectomies using robot- assisted systems in 2012. At our hospital, a robotic surgery committee was formed, and Professor Horie of the Department of Urology began performing robotic prostate cancer surgery in 2013. Having never seen robotic surgery for CRC before, I visited Professor Hanai at Fujita Health University. The operating table was covered with a robotic patient cart, and the instruments connected to the thick robotic arms moved according to the surgeon's actions. Sometimes, the instruments interfered with each other and stopped moving. I was uneasy about my ability to master the surgical technique, but I proceeded with preparations to introduce robotic surgery as a new approach. Because robotic surgery for CRC was not covered by insurance, we first obtained IRB approval. Associate Professor Kojima and I then underwent offsite training on the da Vinci surgical system and obtained the certification.

Next, we visited the Shizuoka Cancer Center twice with a team of surgical nurses and clinical engineers to observe robotic surgery and learn about actual surgical procedures for rectal cancer surgery. Our first case was performed in May 2015^27)^. Professor Kouichi Hanai from Fujita Medical University was present as the proctor, and the surgery was performed successfully. Approximately two years elapsed from the planning stage to clinical implementation. In 2018, the government insurance covered 12 surgical procedures, including rectal cancer surgery. In the same year, the da Vinci Xi system became available and we began performing robotic LLND. Additionally, surgery for colon cancer will be approved by the government insurance coverage in 2022. Our hospital obtained facility certification for colon cancer surgery in 2023, which enabled us to perform all robotic surgeries for colorectal cancer.

**Figure 11:**
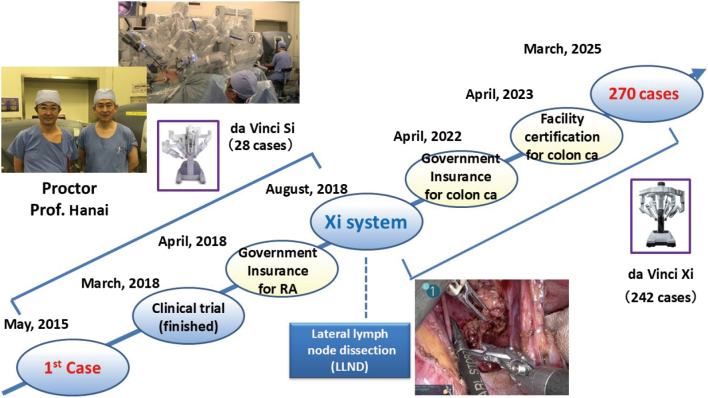
Steps for introducing robotic surgery in our department

As of Mar. 2025, we performed 270 robotic surgeries. Additionally, we obtained experience with 48 cases of ISR and 135 cases of LLND using minimally invasive surgery, including laparoscopic and robotic surgeries (Figure 11).

### Clinical trial on robotic surgery for rectal cancer

Several clinical trials have evaluated the superiority of robotic surgery over laparoscopic surgery. The ROLARR trial^28)^ was a randomized controlled trial (RCT) conducted on 471 rectal cancer cases. The trial evaluated factors such as the conversion rate to open surgery, positive pathological circumferential resection margin (CRM) rate, postoperative complication rate, patient-reported bladder function, and patient-reported sexual function. However, this study failed to demonstrate the superiority of robot-assisted surgery over laparoscopic surgery, including the primary endpoint of the conversion rate to open surgery. In contrast, the REAL trial conducted in China^29)^ reported short-term results. The CRM positive, intraoperative complication, and anastomotic leakage rates were all significantly lower after robotic surgery. In our department, we conducted a retrospective comparison of postoperative urinary dysfunction in 100 patients who underwent LLND for RC but found no difference in urinary dysfunction after LLND between LS and RALS^30)^.

This multicenter prospective observational study of robotic surgery was conducted in Japan. At the same time, it became apparent that pathological examination using a circular incision was not widely used for CRM evaluation of RC in Japan. With the cooperation of Professor Yao from the Department of Pathology, we participated in the PRODUCT trial^31)^ to evaluate CRM in minimally invasive surgeries. Of the 303 patients in this trial, 167 (55%) underwent laparoscopic surgery, and the CRM positivity rate was 8.6%. Next, we conducted a study of 321 robotic surgery cases (VITRUVIANO trial)^32)^, which had a CRM positivity rate of 6.6%. This indicates that robotic surgery yields better outcomes.

We expect to demonstrate the superiority of robotic surgery in the future.

## Conclusion

**Figure 12:**
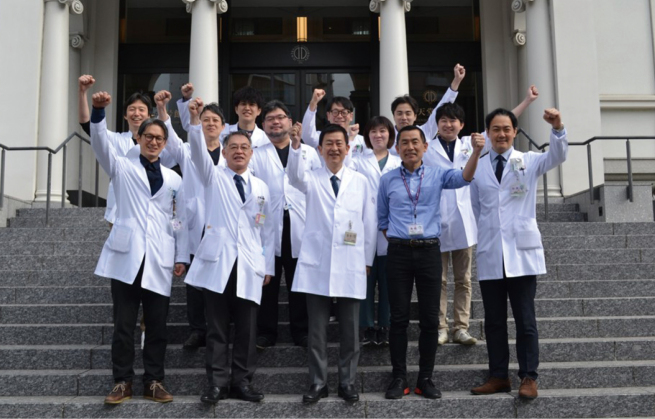
Medical staff in the Department of Coloproctological Surgery, Juntendo University Hospital

During my 41 years as a gastroenterological surgeon at Juntendo University, I have had the privilege of helping establish two minimally invasive surgical techniques: laparoscopic and robotic surgery. These experiences have been invaluable to me. I am deeply grateful to the medical staff in our department (Figure 12) and faculty members at Juntendo University for their support and guidance that enabled me to gain this experience.

## Author contributions

The author wrote the whole manuscript, read and approved the final manuscript.

## Conflicts of interest statement

The author declare that there are no conflicts of interest.
